# Systematic review and meta-analysis of spinal versus general anesthesia in decompressive surgeries of the lumbar spine

**DOI:** 10.1016/j.bas.2025.104280

**Published:** 2025-05-13

**Authors:** Clara F. Weber, Anton Früh, Claudius Jelgersma, Ahmad Almahozi, Kiarash Ferdowssian, Nils Hecht, Peter Vajkoczy, Lars Wessels

**Affiliations:** a Department of Neurosurgery, Charité – Universitätsmedizin Berlin, Charitéplatz 1, 10117, Berlin, Germany; b BIH Biomedical Innovation Academy, BIH Charité Junior Digital Clinician Scientist Program, Anna-Louisa-Karsch-Straße 2, 10178, Berlin, Germany

**Keywords:** Lumbar spine surgery, Decompressive surgery, Local anesthesia, General anesthesia

## Abstract

**Introduction:**

Decompressive lumbar spine surgery is a common procedure for disc herniation and spinal stenosis. Besides intervention under general anesthesia (GA), awake surgery (AS) in local or spinal anesthesia offers potential benefits regarding GA-related side effects and simplified periprocedural management.

**Research question:**

Within this systematic analysis, we sought to compare postsurgical outcomes of spinal decompression surgeries in GA and AS.

**Methods:**

Following the PRISMA guidelines, we extracted all relevant studies from three databases and collected all data concerning surgery duration, blood loss, postoperative duration of hospitalization, postoperative pain (VAS), and disability indices (ODI).

**Results:**

In total, we identified 11 studies covering 1350 patients. AS was associated with shorter surgery duration [Mean difference (MD) −8.52 (95 % confidence interval (CI) −14.56, −2.49) min] as well as lower relative risk for postoperative complications [risk ratio (RR) 0.86 (0.75, 0.99)] and nausea and vomiting [RR 0.58 (0.51, 0.66)]. There were no significant differences in estimated blood loss [MD -27.59 (−61.85, −9.97) ml], hospital stay duration [MD -1.6 (−3.95, 0.75) d], pain [MD -0.22 (−1.35, 0.92) VAS] and disability scales [MD -0.8 (−3.54, 1.94) ODI]. Selected studies were considerably heterogeneic (*I*^*2*^ = 0–99.89 %).

**Discussion and conclusion:**

Awake surgery is a safe and promising alternative to GA in decompressive spine surgery, however, high heterogeneity of the present literature warrant confirmation in future prospective, randomized trials.

## Highlights

• Awake surgery is associated with shorter surgery duration, and decreased risk for postoperative vomiting and nausea and postoperative complications, compared to general anesthesia for decompressive surgery of the lumbar spine.
• Results refine previous evidence for a more closely defined patient cohort and intervention type.
• High heterogeneity measures across studies highlight the need for future prospective, randomized studies with detailed and uniform outcome reporting.

## 1 Introduction

Stenotic pathologies of the spine are increasingly prevalent and encompass a wide array of conditions, most commonly disc herniation and degenerative spinal stenosis (Truumees, 2005)– (Katz et al., 2022). Taken together, they are characterized by compression of neurovascular structures in the spinal canal, provoking debilitating symptoms including pain, claudication, and radiculopathy (Lurie and Tomkins-Lane, 2016), (Schwarzer et al., 1995). As such, stenotic spine conditions impact patients’ mobility, societal participation and ability to work, thus posing a considerable disease burden (Zhou et al., 2024), (Kim et al., 2018) and motivating the efforts to continuously refine therapeutic algorithms.

Currently, treatment approaches depend on several factors including the underlying pathomechanisms, and severity and onset of functional impairment (Lurie and Tomkins-Lane, 2016), (Katz et al., 2022). Decompressive surgery forms a cornerstone in the therapeutic management of these conditions, combined with physical therapy and pain management (Katz et al., 2022), (Kreiner et al., 2014). Surgical intervention aims at the timely decompression of the spinal cord to allow for spatial expansion, ultimately restoring vascular supply and neuronal signal transmission (Karlsson et al., 2022), (Phillips and Cunningham, 2002). Surgical techniques differ depending on the site and mechanism of stenosis but commonly encompass laminectomy (Truumees, 2005), (Lurie and Tomkins-Lane, 2016), potentially combined with discectomy (Kreiner et al., 2014), (Postacchini and Postacchini, 2011), to alleviate physical stress on underlying nerves. Intervention can necessitate additional stabilization through spondylodesis, *e.g.,* to address segmental instability or in cases requiring the removal of a critical amount of excess bone (Dower et al., 2016).

While spinal decompression surgeries are routinely performed and considered safe procedures, patients are at risk for post-surgical pain exacerbation and potential side effects from anesthesia (Turel and Bernstein, 2016), (Garg et al., 2022). One promising method to modulate these factors is the administration of spinal anesthesia and awake surgery, thereby avoiding general anesthesia and its associated complications (Mooney et al., 2022), (Rajjoub et al., 2024). Previous evidence has suggested potential benefits of this technique, highlighting a decrease in periprocedural adverse effects, such as nausea, delirium, as well as an expedited recovery time (Garg et al., 2022), (Rajjoub et al., 2024), (Meitzen and Black, 2023). In particular, in an aging society, the risk profile for side effects from general anesthesia increased. Thus, spinal decompression through awake surgery could remain accessible for this vulnerable patient group.

In this study, we aim to provide a systematic literature review and meta-analysis for spinal versus general anesthesia in decompressive spinal surgery not requiring spondylodesis by conducting an in-depth structured literature review and analysis according to the PRISMA guidelines (Page et al., 2021). Furthermore, we aim to provide evidence for the best periprocedural management for patients opting for surgical intervention, specifically in conditions requiring decompressive lumbar surgery.

## 2 Methods

### 2.1 Database search and inclusion criteria

The primary literature search involved initial screening in three major databases (Cochrane Library, Ovid MEDLINE, Ovid Embase) filtering for the following combination of medical subject headings (MeSH) terms: (“Anesthesia, Spinal” [MeSH] OR (“Anesthesia, General” [MeSH] OR Anesthesia, Epidural” [MeSH]) AND (“Spinal Stenosis” [MeSH] OR “Diskectomy” [MeSH] OR “Laminectomy” [MeSH] OR “Decompression, Surgical” [MeSH]). Database search followed the Preferred Reporting Items for Systematic Reviews and Meta-Analyses of Diagnostic Test Accuracy (PRISMA) statement (Page et al., 2021).

We included peer-reviewed studies that (i) involved adult patients ( ≥ 18 years), (ii) compared spinal to general anesthesia in patients who underwent decompressive spine surgery, (iii) reported postoperative outcome measures, and (iv) had full-text articles in English available. Studies were ineligible and excluded from review if they (i) reported outcomes in the form of case reports, reviews and editorials, (ii) reported on inadequate interventions, or (iii) reported on non-human subjects.

### 2.2 Data extraction

In the initial screening process, we used an online systematic review tool, Covidence (Veritas Health Innovation, Melbourne, Australia, available at www.covidence.org). Briefly, all identified articles were screened for relevance and adherence to inclusion criteria by a graduate student (CFW) and a neurosurgical resident (AF). Upon initial abstract screening, full-text articles were assessed and selected by CFW and AF, and confirmed by an experienced board-certified neurosurgeon (LW). Discrepancies were resolved by majority decisions.

For all studies, the year of publication, data collection timespan and sample size were identified. Additionally, the following data points were extracted from full text files for awake surgery (AS) and general anesthesia (GA) subgroups respectively: Surgery duration, number of levels operated, post-operative Oswestry disability index (ODI) (Fairbank and Pynsent, 2000), postoperative pain on a visual analogue scale (VAS), sex distribution, BMI, complications and estimated blood loss.

### 2.3 Statistical analysis

After initial data extraction and quality control, we determined mean differences for all continuous outcomes, *i.e.*, surgery duration, estimated blood loss, postoperative hospital stay, as well as postoperative ODI and pain. To assess the risk of complications, we extracted the number of surgical complications as well postprocedural nausea and vomiting, and subsequently calculated risk ratios between the AS and GA groups, using Fisher's exact test (Fisher, 1992). Risk ratios are depicted with GA as reference group, so that RR > 1 indicates higher risk in the AS group and RR < 1 lower risk in the AS cohort. Outcomes were visualized in forest plots and heterogeneity across selected studies was quantified as I^2^, a measure quantifying the nonrandom variation among studies and typically indicating high heterogeneity at I^2^>50 %.

We used the methodological index for non-randomized studies (MINORS) to assess risk of bias (Slim et al., 2003). This tool lists eight items for non-comparative research, along four additional categories for comparative studies. Within each category, every study is assigned zero (not reported), one (reported but inadequate) or two (adequately reported). As such, comparative and non-comparative studies can reach a maximum of 24 or 16 points respectively, with a cutoff of ≥12 and ≥ 8 points indicating satisfactory quality (Slim et al., 2003). Data analysis was conducted in R v. 4.3.1 (R Core Team, 2020), using the *metafor* package (“The metafor Package).

## 3 Results

### 3.1 Selected studies

**Fig. 1:**
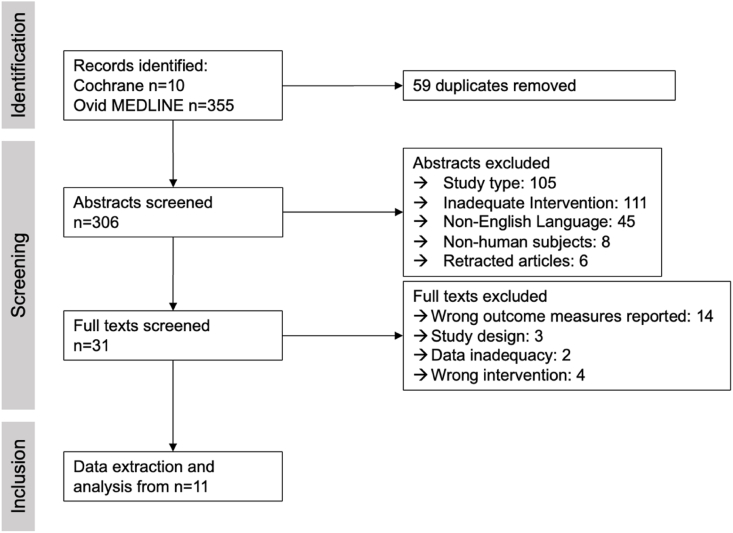
**Study selection** Flowchart of study selection according to the PRISMA statement.

A total of 365 studies were identified in the initial search. After initial abstract and full-text screening, 59 and 275 were excluded respectively, concluding a final sample size of eleven studies, of which nine compared AS to GA and two studies elaborated on AS in isolation (see Fig. 1). Collectively, these studies entailed data of n = 1350 patients with a male-to-female ratio of 747:603 (55:45 %), including 688 (51 %) patients who underwent awake surgery and 662 (49 %) who received general anesthesia. Mean age ranged from 40.2 years to 82.1 years and mean BMI was between 23.8 and 30.26 kg/m^2^. More detailed patient characteristics of the included patients are provided in Table 1. All study cohorts included both female and male participants, with the percentage of female patients ranging between 22 % and 60 %. Cohorts were relatively heterogeneous regarding age, spanning from mean age of 40.2–82.1 years in the AS group, while age distribution within AS and GA arms within studies appeared more balanced (see Table 1). Perioperative risk was comparable across most studies, as most studies included American Society of Anesthesiologists (ASA) physical status classes I-III, except for three studies that did not include I and II respectively, and one cohort which included 3 ASA IV patients.

**Table 1:** Demographic characteristics of the patients of the included studies. AS = awake surgery, ASA = American Society of Anesthesiologists perioperative risk assessment, BMI = body mass index, GA = general anesthesia, SD = standard deviation.

Author	n (AS)	% female (AS)	Age AS:GA (mean ± SD or median) [y]	BMI or weight AS: GA (mean ± SD) [kg/m^2^ or kg]	Range spinal levels	ASA range
Dagistan et al., 2014	180 (90)	29 % (32 %)	42:38	NA:NA	1	NA
Dashtbani et al., 2019	72 (36)	49 % (44 %)	43.1 ± 14.1:46.2 ± 13.8	NA:NA	1	I-II
Guan et al., 2019	120 (60)	60 % (57 %)	74.2 ± 12.6:73.8 ± 13.1	55.2 kg ± 6.8 kg:55.7 kg ± 7.1 kg	1	NA
Jellish et al., 1996	122 (61)	46 % (43 %)	43 ± 2:46 ± 2	84.3 kg ± 2.4 kg:84.9 kg ± 2.3 kg	1–2	I-III
Kindris et al., 2023	154 (56)	51 % (52 %)	82.1 ± 3.25:79.9 ± 4.25	26.2 ± 5.025:27.9 ± 5.775	1–3	I-IV
McLain et al., 2005	400 (200)	41 % (36 %)	48:47	NA:NA	1–2	I-III
Papadopoulos et al., 2006	43 (27)	50 % (NA)	40.2 ± 9.8:34.7 ± 7.7	74.1 kg ± 15.3 kg:72.6 kg ± 11 kg	1	NA
Sadrolsadat et al.*,* 2008	100 (50)	50 % (50 %)	45.7 ± 5.2:45.2 ± 5.6	77.8 kg ± 7.5 kg:75.2 kg ± 8.2 kg	1	I-III
Tarikci Kilic et al.*,* 2018	101 (50)	50 % (48 %)	54.1 ± 2.4:53.2 ± 3.1	27.33 ± 3.69:29.15 ± 5.37	1	I-III
Akakin et al., 2015	27 (27)	22 % (22 %)	60.04 ± 10.639:NA ± NA	30.26 ± 4.37:NA	NA	NA
Wu et al., 2024	31 (31)	58 % (58 %)	74.09 ± 6.93:NA ± NA	23.8 ± 5.71:NA	1–2	II-III

Duration of surgery and length of stay is provided in Table 2. In the identified studies, awake surgery was mostly conducted using spinal anesthesia achieved application of a bupivacaine solution in the subarachnoid space, additionally, three studies administered epidural anesthesia. Table 3 outlines the exact dosage, substance and volume of anesthestics used respectively.

**Table 2:** Study characteristics. AS = awake surgery, GA = general anesthesia, SD = standard deviation.

Author	Timespan	Surgery duration AS:GA (Mean ± SD) [min]	Hospital stay AS:GA (Mean ± SD) [min]	Estimated bloodloss AS:GA (Mean ± SD) [ml]
Dagistan et al.*,* 2014	2012–2013	71 ± 12:85 ± 15	NA	246 ± 8.4:275 ± 8.8
Dashtbani et al., 2019	2016–2019	64.7 ± 16.1:70 ± 18.1	NA	55.8 ± 23.6:62.2 ± 30.6
Guan et al., 2019	2016–2017	47.6 ± 15.7:45.4 ± 15.5	3 ± 0.5:7 ± 0.5	7 ± 2:7 ± 2.5
Jellish et al., 1996	NA	67.1 ± 2.8:81.5 ± 3.6	NA	133 ± 13:221 ± 32
Kindris et al., 2023	2019–2020	60±NA:83±NA	5.1:6.3	76:120
McLain et al., 2005	1994–1998	105 ± 15:120 ± 15	NA	NA
Papadopoulos et al., 2006	2005	65.4 ± 15.2:63.6 ± 26.6	2.1:1.8	NA
Sadrolsadat et al.*,* 2008	2005–2007	94.4 ± 17.3:94.1 ± 17.9	NA	464.5 ± 69.3:438 ± 66.6
Tarikci Kilic et al.*,* 2018	NA	103.2 ± 29.4:127.8 ± 50.4	1 ± 0.16:1.5 ± 0.6	96.3 ± 47.41:164.2 ± 90.47
Akakin et al., 2015	2012–2013	45.56 ± 10.86:NA	NA	NA
Wu et al., 2024	2021–2022	62.85 ± 30.4:NA	2.9 ± 1.48:NA	NA

**Table 3:** Anesthesia in awake group, substances and dosages used as documented in original studies. AS = awake surgery, GA = general anesthesia.

Author	Anesthesia in AS group	Dosage and Administration	Complications AS
Dagistan et al.*,* 2014	Spinal	3 ml 0.75 % bupivacaine, single injection L3/L4 intervertebral space	Urinary retention (7 GA *vs.* 23 AS), pneumonitis (5 GA *vs.* 1 AS), discitis (3 GA *vs.* 1 AS), wound infection (2 GA *vs.* 1 AS), CSF fistula (0 GA *vs.* 5 AS)
Dashtbani et al., 2019	NA	NA	Incidental dural tear, superficial wound infection
Guan et al., 2019	Epidural	3–5 ml 1.33 % lidocaine	NA
Jellish et al., 1996	Spinal	11 mg bupivacaine L4/5 interspace	Urinary retention (14.8 % GA *vs.* 22.9 % AS)
Kindris et al., 2023	NA	NA	Incidental dural tear (3 in GA *vs.* 1 in AS), hematoma requiring revision (2 GA *vs.* 1 in AS), postoperative delirium (6 GA *vs.* 2 AS), urinary retention
McLain et al., 2005	NA	NA	Urinary retention (46 GA *vs.* 16 AS), spinal headache (6 GA *vs.* 3 AS), pulmonary dysfunciton (3 GA *vs.* 2 AS)
Papadopoulos et al., 2006	Epidural	20–30 ml of 2 % lidocaine	NA
Sadrolsadat et al.*,* 2008	Spinal	4 ml of 0.5 % bupivacaine	NA
Tarikci Kilic et al.*,* 2018	Spinal	3 ml of 0.5 % bupivacaine	NA
Akakin et al., 2015	Epidural	20–30 ml of 2 % lidocaine with epinephrine (1:200.000) +100 μg fentanyl	NA
Wu et al., 2024	NA	NA	Intraoperative neck pain, transient postsurgical lower limb numbness

### 3.2 Postoperative outcomes and complications

**Fig. 2:**
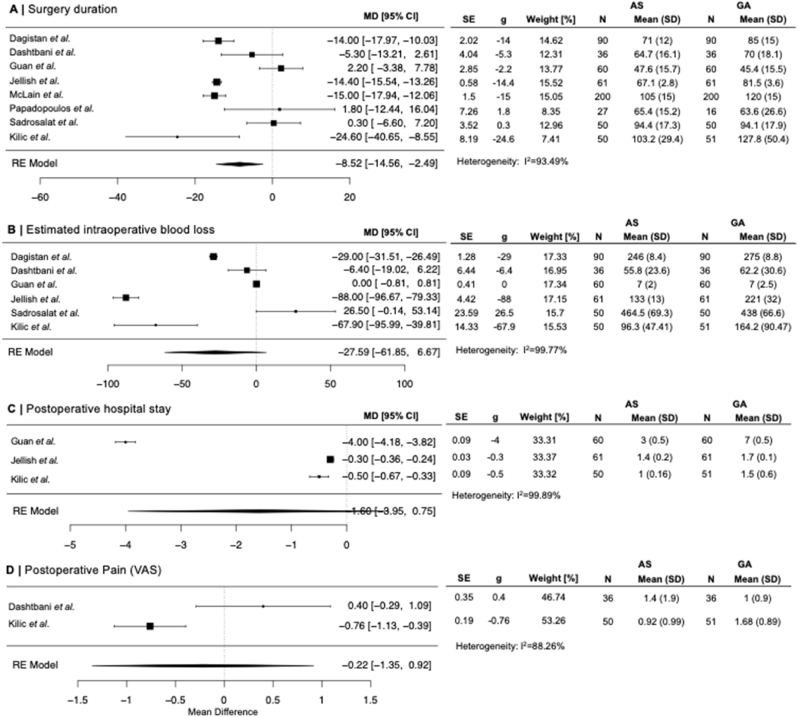
**Forest plots** Forest plot of surgery duration, postoperative hospital stay duration, estimated blood loss, and postoperative visual analogue pain scale as mean difference between the AS and GA group.

AS was associated with lower mean operative time [overall mean difference (MD) −8.53 (95 % confidence interval (CI): −14.56, −2.49) minutes]. Both intraoperative blood loss and postoperative hospital stay were reduced in the AS group, albeit not statistically significant [MD -27.59 (95 % CI: −61.85, −9.97) ml, MD -1.6 (95 % CI: −3.95, 0.75) d, respectively]. Fig. 2 depicts the mean differences in operating time and estimated blood loss as well as post-operative hospital stay between all studies in which such data was available for both groups. Mean differences in post-operative pain as assessed on a visual analogue scale between the AS and GA groups did not reach statistical significance [MD -0.22 (95 % CI: −1.35, 0.92) VAS], presented in Fig. 2.

**Fig. 3:**
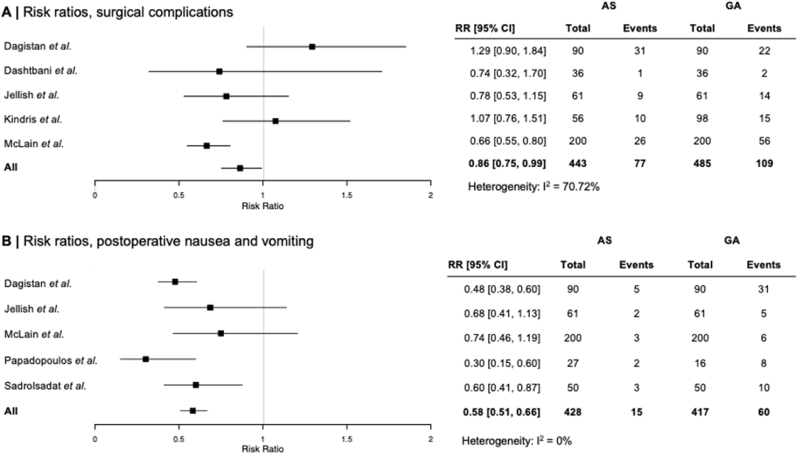
**Risk ratios** Risk ratios of overall surgical complications as well as postoperative nausea and vomiting between the AS and GA groups.

AS was favorably associated with less postoperative complications at a risk ratio of 0.86 (95 % CI: 0.75, 0.99). Complications included incidental dural tears, wound infections and hematoma requiring surgical revision. No Clavien-Dindo class four or five complications (Dindo et al., 2004) were reported in the selected studies. An exhaustive list of postsurgical complications as described in original articles is outlined in Table 3. We additionally assessed the occurrence of postoperative nausea and vomiting, which appeared advantageous in the AS group at a risk ratio of 0.58 (95 % CI: 0.51, 0.66). Risk ratios of overall surgical complications as well as postoperative nausea and vomiting between the AS and GA groups are presented in Fig. 3.

### 3.3 Bias and heterogeneity

Of note, there was considerable heterogeneity across selected studies, with I^2^ measures ranging between 0 % and 99.89 %, with highest heterogeneity reported in analysis of postoperative hospital stay (I^2^ = 99.89 %) and blood loss (I^2^ = 99.77 %) measures.

Risk of bias as assessed using the MINORS tool is reported in Table 4. Mean scores culminated to a mean of 15.9 ± 1.4, thus reaching a satisfactory threshold.

**Table 4:** Risk of Bias assessment. RoB2 = Risk of Bias assessment, ROBINS-E = Risk Of Bias In Non-randomized Studies - of Exposures.

Study	Clearly stated aim	Consecutive patient inclusion	Prospective data collection	Appropriate endpoints	Unbiased assessment of endpoint	Appropriate follow-up period	Loss to follow up <5 %	Prospective study size calculation	Control group adequacy	Contemporary groups	Baseline equivalence	Adequate statistical analyses
Dagistan et al.*,* 2014	2	0	1	2	1	2	0	0	2	2	2	2
Dashtbani et al., 2019	2	0	2	2	2	2	0	0	2	2	0	2
Guan et al., 2019	1	2	2	2	2	2	0	0	2	2	0	2
Jellish et al., 1996	1	0	2	2	2	2	0	0	2	2	2	2
Kindris et al., 2023	2	0	1	2	2	2	0	0	2	2	2	2
McLain et al., 2005	2	2	1	2	2	2	0	0	2	2	2	2
Papadopoulos et al., 2006	1	1	2	2	2	2	0	0	2	2	2	2
Sadrolsadat et al.*,* 2008	2	2	2	2	2	2	0	0	2	2	2	2
Tarikci Kilic et al.*,* 2018	2	1	2	2	2	2	0	0	2	2	2	2
Akakin et al., 2015	2	2	2	2	1	2	0	0	na	na	na	na
Wu et al., 2024	1	2	0	2	1	2	0	0	na	na	na	na

## 4 Discussion

The main finding of this analysis is that outcomes of lumbar decompressive surgery in AS are comparable to GA. AS was associated with shorter surgery duration and decreased risk for complications as well as postoperative nausea and vomiting, while there was no difference in estimated intraoperative blood loss, postoperative hospital stay, as well as postoperative pain and disability outcomes.

There is an increasing prevalence of conditions requiring decompressive lumbar spine surgery. However, the potential risks associated with general anesthesia especially for elderly patients motivate the effort for this systematic review to illustrate the current literature concerning postoperative outcomes for awake decompressive spine surgery *vs.* under general anesthesia. Following the PRISMA guidelines (Page et al., 2021), we have identified eleven studies that were included in this review, of which nine compared GA to AS, and two studied awake decompressive surgery in isolation. We found that AS was associated with shorter postoperative hospital stay and lower postoperative complication rates. Overall, awake surgery was associated with favorable outcomes regarding blood loss, postoperative pain, and overall complication burden, while surgery duration, postoperative pain and disability indices were not favorably influenced by awake surgery.

Decompressive surgery is a common and safe procedure constituting a central therapeutic approach in the treatment of stenotic spine conditions (Lurie and Tomkins-Lane, 2016), (Kreiner et al., 2014), and traditionally requires general anesthesia. This poses a considerable challenge to the cardiovascular and nervous system (Meitzen and Black, 2023), (Roy, 2000), (Wang et al., 2022). Specifically, studies have pointed out the higher prevalence of delirium and longer post-surgical hospital stay associated with general anesthesia (De Biase et al., 2022), (Finsterwald et al., 2018), which are especially common among elderly patients undergoing GA (Lessing et al., 2017)– (Wu et al., 2024), thus motivating efforts to prevent prolonged anesthesia. AS has been proposed in numerous previous studies as a promising opportunity to circumvent anesthesia-related postsurgical complications (Garg et al., 2022), (Rajjoub et al., 2024), and has thus far proven safe and effective(Akakin et al., 2015; Dashtbani et al., 2019; McLain et al., 2005).

In the articles investigated in the present review, AS was associated shorter surgery duration, thus hinting towards a benefit of AS in patients that are likely to experience side effects from prolonged GA (Guan et al., 2019), (Kindris et al., 2023). Apart from an advantageous safety and post-anesthesia deficit profile, shorter surgery times also contribute to more time efficiency and overall productivity for the surgical departments in question (Kilic and Naderi, 2019). Thus, AS can also contribute to shorter turnover times, ultimately contributing to higher surgery capacities and better surgical care for all patients awaiting decompressive surgery (Finsterwald et al., 2018), (Olmos et al., 2023).

Moreover, AS was associated with lower risk for complications such as wound infections, thus potentially shrinking the need for revision surgery (Kindris et al., 2023). A similar effect could emerge from finding of reduced rates of postoperative nausea and vomiting, hence potentially decreasing the need for prolonged admission to post-anesthesia care units and thus increasing overall efficiency (Kilic and Naderi, 2019), (Sadrolsadat et al., 2009). Of note, similar aspects of AS have been studied in other conditions such as hip arthroplasty that can be performed under GA as well as in AS, with similar outcomes pointing to the overall advantages of AS (Bhushan et al., 2022), (Neuman et al., 2021), (Kurtoğlu et al., 2009).

In a recent study, Rajjoub et al. have provided a comprehensive review of postoperative outcomes for spinal surgery between patients who underwent spinal vs general anesthesia, which showed slightly favorable outcomes concerning recovery times and postoperative complications associated with spinal anesthesia (Rajjoub et al., 2024), (Jellish et al., 1996). This work has provided a robust foundation for continued research to explore the large-scale feasibility of awake surgery in spinal procedures. In this review, we defined a narrower target patient collective, specifically those undergoing decompressive spinal surgery for treatment of spinal stenosis and/or herniated nucleus pulposus without additional stabilization through spondylodesis. Solely decompressive surgical approaches differ from stabilization in several aspects. These include less extensive tissue manipulation as well as a narrowed risk profile for infections, as no foreign material is inserted (Katz et al., 2022), (Karlsson et al., 2022), (Shen et al., 2021). Another important aspect is the tolerability of awake surgery without spondylodesis as it is less invasive, associated with shorter surgery times (Karlsson et al., 2022), and thus potentially psychologically better to process for affected patients (Fiani et al., 2021), (De Biase et al., 2022).

### 4.1 Strength and limitations

In this study, we have provided a structured review and meta-analysis of all relevant and eligible evidence comparing spinal to general anesthesia in spinal stenosis surgery cases following the PRISMA statement (Page et al., 2021). One strength of this study is the summary of multi-centric and heterogeneous data, implicating the coverage of a broad patient collective. However, high heterogeneity measures in continuous outcome measures imply a substantial limitation to the comparability across studies and thus limit the statistical robustness of pooled results. To contextualize this finding, we additionally provide visualizations of effects for individual studies. Moreover, while the anesthesia protocols were comparable and similar, they were not identical regarding administration, dosage, and the medications used. Occasionally, small doses of systemic propofol are administered even during spinal anesthesia, which can lead to nausea and delirium. While we acknowledge this as a caveat to the present study, we see a potential benefit in the presented evidence across different anesthesiologic protocols. In future prospective studies, clear anesthesia protocols should be defined to ensure greater comparability and reproducibility of the results.

One important limitation is the heterogeneity of study populations in terms of modality and severity of stenosis. The variety of stenotic conditions investigated here requires different surgical approaches with variable tissue manipulation and associated risks. With an increasing number of affected levels, calcifications and stenosis severity, risk profiles become increasingly complex, mitigating postoperative outcomes. In this report, we sought to narrow surgery indications compared to previous reviews, while acknowledging the remaining uncertainty as a limitation to the generalizability of our results. All studies laid out in this review outlined outcomes from lumbar spine surgery, thus our results do not reflect on higher spinal levels. However, given that stenosis is most prevalent in the lumbar spine (Guan et al., 2019), our results are nonetheless of clinical interest.

Of note, given the variation in underlying study protocols, postoperative outcome measures were unavailable for some of the studies (Guan et al., 2019), (Akakin et al., 2015), (Sadrolsadat et al., 2009), (Papadopoulos et al., 2006), which limited the analysis of postsurgical outcomes. Additionally, heterogeneity measures were considerably high across the selected studies, which we acknowledge as an important caveat to our results. Furthermore, most studies included were conducted retrospectively with a notable lack of randomized prospective trials available to date, posing an acknowledgeable publication bias (Table 4). In conclusion, further, large prospective trials are necessary to ultimately assess the benefits of awake decompressive surgery.

## 5 Conclusions

In lumbar decompressive spine surgery AS was compared to GA associated with shorter surgery duration and decreased risk for complications as well as postoperative nausea and vomiting, while there was no difference in estimated intraoperative blood loss, postoperative hospital stay, as well as postoperative pain and disability outcomes. Overall, AS thus proved safe and effective, while being beneficial in terms of surgery duration and complications, and not inferior to GA in secondary outcome measures. However, the limited number of comparable studies and high heterogeneity of the present literature warrant confirmation in future prospective, randomized trials with clear anesthesia protocols.

## Authorship contribution

Idea: LW, PV, NH; literature search and data analysis: CFW, AF, LW; manuscript draft: CFW, AF; critical revision and methodological input: CJ, AA, KF, NH.

## Funding sources

This research did not receive any specific grant from funding agencies in the public, commercial, or not-for-profit sectors.

## Declaration of competing interest

The authors declare that they have no known competing financial interests or personal relationships that could have appeared to influence the work reported in this paper.
